# Porcine Epidemic Diarrhea Virus Induces Autophagy to Benefit Its Replication

**DOI:** 10.3390/v9030053

**Published:** 2017-03-19

**Authors:** Xiaozhen Guo, Mengjia Zhang, Xiaoqian Zhang, Xin Tan, Hengke Guo, Wei Zeng, Guokai Yan, Atta Muhammad Memon, Zhonghua Li, Yinxing Zhu, Bingzhou Zhang, Xugang Ku, Meizhou Wu, Shengxian Fan, Qigai He

**Affiliations:** 1 State Key Laboratory of Agricultural Microbiology, College of Veterinary Medicine, Huazhong Agricultural University, Wuhan 430070, China; guoxiaozhen@webmail.hzau.edu.cn (X.G.); zhangmengjia@webmail.hzau.edu.cn (M.Z.); 632071039@webmail.hzau.edu.cn (X.Z.); wstx1992@163.com (X.T.); 15071194245@163.com (H.G.); aiyouwei0620@163.com (W.Z.); Memonatta80@webmail.hzau.edu.cn (A.M.M.); lzh1990@webmail.hzau.edu.cn (Z.L.); yingzizhu10@163.com (Y.Z.); abing0313@webmail.hzau.edu.cn (B.Z.); kuxugang84@163.com (X.K.); wumeizhou@mail.hzau.edu.cn (M.W.); 2 The Cooperative Innovation Center for Sustainable Pig Production, Wuhan 430070, China; gkyangk@163.com; 3 College of Animal Sciences and Technology, Huazhong Agricultural University, Wuhan 430070, China

**Keywords:** autophagy machinery, PEDV replication, inflammatory responses, apoptosis

## Abstract

The new porcine epidemic diarrhea (PED) has caused devastating economic losses to the swine industry worldwide. Despite extensive research on the relationship between autophagy and virus infection, the concrete role of autophagy in porcine epidemic diarrhea virus (PEDV) infection has not been reported. In this study, autophagy was demonstrated to be triggered by the effective replication of PEDV through transmission electron microscopy, confocal microscopy, and Western blot analysis. Moreover, autophagy was confirmed to benefit PEDV replication by using autophagy regulators and RNA interference. Furthermore, autophagy might be associated with the expression of inflammatory cytokines and have a positive feedback loop with the NF-κB signaling pathway during PEDV infection. This work is the first attempt to explore the complex interplay between autophagy and PEDV infection. Our findings might accelerate our understanding of the pathogenesis of PEDV infection and provide new insights into the development of effective therapeutic strategies.

## 1. Introduction

The new porcine epidemic diarrhea (PED) outbreaks caused by porcine epidemic diarrhea virus (PEDV) variant has been documented in China since late 2010 and is now distributed all over the world. PED is characterized by acute enteric infection and high mortality in sucking piglets, causing enormous economic losses to the swine industry [1,2,3,4]. PEDV is an enveloped, single-stranded positive-sense RNA virus of the Coronaviridae family. The viral genome is approximately 28 kb, arranged with at least seven open reading frames (ORFs), ORF1a, ORF1b, S, ORF3, E, M, and N. ORF1a and ORF1b are further processed into 16 nonstructural proteins, nsp1 to nsp16. The S, E, M, and N genes encode four structural proteins, whereas ORF3 encodes an accessory protein [5,6,7]. Despite the elucidation of PEDV pathogenesis in some aspects, the underlying mechanism of PEDV replication is still largely unknown.

Autophagy is an evolutionarily highly conserved intracellular degradation process in which double-membrane vesicles (termed autophagosomes) are generated, and the long-lived proteins and damaged organelles are delivered to lysosomes for degradation and recycling [8,9]. Autophagy can be induced by diverse intracellular and extracellular stimuli, such as nutrient starvation, endoplasmic reticulum (ER) stress, pathogen-associated molecular patterns (PAMPs), and virus infection [10]. Increasing evidence indicates that autophagy plays both anti-viral and pro-viral roles in the life cycles and pathogenesis of a broad range of viruses [11]. Specifically, autophagy is an intrinsic host defense mechanism that inhibits viral replication or eliminates viruses by delivering them to the lysosomal compartment for degradation. Meanwhile, viruses develop many mechanisms to block autophagy or even hijack it for their own benefit, such as human cytomegalovirus (HCMV) and herpes simplex virus type 1 (HSV-1) [9,12,13,14]. However, autophagy is also believed to serve as a platform for viral replication, especially for RNA viruses, such as classical swine fever virus (CSFV), porcine reproductive and respiratory syndrome virus (PRRSV), and rotavirus (RV), utilizing the membranes of the autophagosome-like vesicles for their replication [15,16,17]. These polar characteristics reveal the complicated relationship between autophagy and viral infection.

A previous proteomic study indicated that more differentially expressed proteins were mapped to the autophagy pathway, and the microtubule-associated protein 1B, a useful biomarker protein for autophagy was up-regulated in PEDV-infected Vero cells [18]. In addition, our previous study demonstrated that mTOR (the mammalian target of rapamycin) pathway, which was closely associated with cellular autophagy, was down-regulated, and that the autophagy associated protein ATG5 was up-regulated during PEDV infection [19]. These studies indicated that autophagy might participate in PEDV infection, but the specific function of autophagy in the process of PEDV infection has not been elucidated. In the present study, we demonstrated for the first time that autophagy was triggered in Vero cells during PEDV infection to promote its replication. Moreover, autophagy might mediate the inflammatory responses induced by PEDV infection and have a positive correlation with the NF-κB signaling pathway.

## 2. Materials and Methods

### 2.1. Cells and Viruses

African green monkey kidney cell lines, Vero-E6 cells, were cultured in Dulbecco’s modified Eagle’s medium (DMEM), supplemented with 10% fetal bovine serum (Invitrogen, Carlsbad, CA, USA) at 37 °C with 5% CO_2_. The PEDV variant strain CH/YNKM-8/2013 (Accession no. KF761675) was isolated from a sucking piglet with acute diarrhea. To obtain replication-incompetent PEDV, virus suspension was irradiated with UV light for 1 h. The absence of virus infectivity was confirmed by TCID_50_ and real-time PCR [16].

### 2.2. Virus Infection

For autophagy induction and inhibition experiments, Vero cells were pretreated with rapamycin (1 μg/mL, Santa Cruz, CA, USA), 3-methyladenine (3-MA, 5 μm, Sigma, St. Louis, USA), Chloroquine (CQ, 50 μm, Sigma), and BAY 11-7082 (10 μm, Sigma) for the indicated time, and were then infected with PEDV at a MOI (multiplicity of infection) of 0.1. After 1 h incubation at 37 °C, unbound viruses were removed by washing three times with PBS, followed by incubation with serum-free DMEM with 8 μg/mL trypsin (Invitrogen) containing varying concentrations of rapamycin, 3-MA, CQ, BAY 11-7082, or DMSO.

### 2.3. Quantitative Real-Time PCR

Total RNA was extracted from Vero cells using the TRIzol reagent (Invitrogen) according to the manufacturer’s protocol, and was then reverse-transcribed into cDNA using oligo (dT) as the primer (Invitrogen). Relative and absolute quantitative real-time PCR were performed in an Applied Biosystems ViiA 7 real-time PCR system as previously described [19]. The primers and probe used are listed in Table 1.

### 2.4. Transmission Electron Microscopy

Vero cells were mock infected or infected with PEDV at 0.1 MOI and collected at 24 h post-infection (hpi) for ultrastructural analysis. Ultra-thin sections were viewed on a Hitachi H-7650 transmission electron microscope (Hitachi Ltd., Tokyo, Japan). Autophagosome-like vesicles were defined as double- or single-membrane vesicles measuring 0.3 to 2.0 μm in diameter with clearly recognizable cytoplasmic contents.

### 2.5. Confocal Fluorescence Microscopy

Vero cells were seeded on coverslips and transfected with GFP-LC3 or mRFP-GFP-LC3. After transfection for 24 h, the cells were infected with PEDV and fixed with cold 4% paraformaldehyde. After permeabilization and blocking, the cells were then incubated with mouse monoclonal antibody directed against the PEDV S protein (made in our laboratory), and were then inoculated with Alexa Fluor 594 Donkey Anti Mouse IgG (H+L) antibody (Ant Gene). Cell nuclei were counterstained with 0.01% 4’,6-diamidino-2-phenylindole (DAPI, Invitrogen). The fluorescent images were examined under a confocal laser scanning microscope (LSM 510 Meta, Carl Zeiss, Munich, Germany).

### 2.6. Western Blot Analysis

Vero cells were lysed in lysis buffer containing 50 mM Tris-HCl (pH 6.8), 10% glycerol, and 2% SDS [20]. The protein concentration was quantified by the BCA protein assay kit and equal amounts of protein samples were mixed with 5× sample loading buffer and boiled for 10 min, and then separated by 12% sodium dodecyl sulfate polyacrylamide gel electrophoresis (SDS-PAGE). The proteins were electro-transferred to 0.45 μm PVDF membranes (Millipore, Mississauga, ON, Canada). Membranes were blocked with 5% (w/v) skim milk-TBST at room temperature for 2 h and then incubated overnight at 4 °C with primary antibodies. The blots were then incubated with corresponding horseradish peroxidase (HRP) conjugated secondary antibodies (ABclonal, Wuhan, China). The protein bands were visualized using the Clarity™ Western ECL Blotting Substrate (Bio-Rad, Hercules, CA, USA). The protein blots were quantified by Image J software (National Institutes of Health, Bethesda, MD, USA).

### 2.7. RNA Interference

Vero cells grown to 60% confluence were transfected separately with Beclin1 or ATG5 and the corresponding scrambled siRNA with Lipofectamine 2000 (Invitrogen) according to the manufacturer’s guidelines. The silencing efficiency was determined by Western blot and real-time PCR. Twenty-four hours after transfection, the cells were infected with PEDV as described above.

The siRNA was designed by and obtained from GenePharma (Shanghai, China). *Beclin1* siRNA sequence: 5’-CCCAGUGUUCCCGUAGAAUTTAUUCUACGGGAACACUGGGTT-3’; *ATG5* siRNA sequence: 5’-GCAACUCUGGAUGGGAUUATTUAAUCCCAUCCAGAGUUGCTT-3’.

### 2.8. Cell Viability Assay

The cytotoxic effects of reagents on Vero cells were determined using the MTT (3-[4, 5-dimethylthiazol-2-yl]-2, 5-diphenyl-2H-tetra-zolium bromide) assay as previously described [21]. Briefly, Vero cells were inoculated in a 96-well plate and treated with different concentrations of pharmacological drugs. Then, the cells were inoculated with MTT and the resulting formazan crystals were dissolved in dimethyl sulfoxide (DMSO). The absorbance was measured by a microplate spectrophotometer at a wavelength of 490 nm.

### 2.9. Statistical Analysis

All experiments were performed independently three times, and variables are expressed as the means with SEM. Statistical analyses were performed using student’s *t*-test. A *p*-value < 0.05 was considered as statistically significant.

## 3. Results

### 3.1. PEDV Infection Increases the Levels of Autophagy in Vero Cells

**Figure 1:**
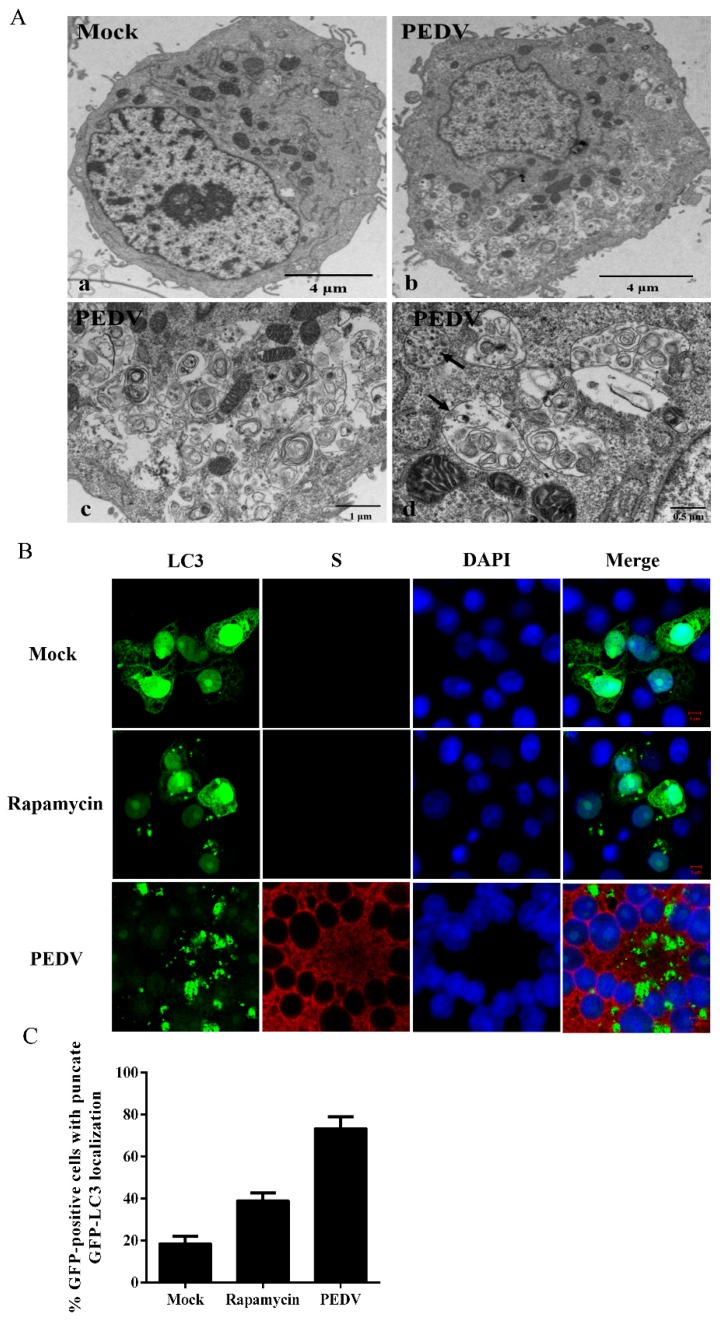
Porcine epidemic diarrhea virus (PEDV) infection increases the formation of autophagosome-like vesicles. (**A**) TEM observation. Vero cells were mock-treated (**a**) or infected with PEDV at 0.1 MOI for 24 h (**b**). Scale bar, 4 μm (**a**,**b**). (**c**) higher-magnification views of (**b**). Scale bar, 1 μm. (**d**) enlargement of the autophagosome-like structure. Scale bar, 0.5 μm. (**B**) Confocal microscope. The redistribution of GFP-LC3 was induced by PEDV infection. Vero cells were transfected with the plasmid GFP-LC3. Twenty-four hours later, the transfected cells were infected or mock-infected with PEDV at 0.1 MOI for 18 h. Meanwhile, cells pretreated with rapamycin for 4 h served as a positive control. PEDV infection was detected with the monoclonal antibody against PEDV S and cell nuclei were counterstained with 4',6-diamidino-2-phenylindole (DAPI). Scale bar, 5 μm. (**C**) The relative number of cells with punctate GFP-LC3 locations relative to all green fluorescent protein-positive cells. The data were presented as mean ± SEM of three independent experiments.

Whether PEDV infection can activate the autophagy machinery was investigated by examining the formation of autophagosome-like vesicles in Vero cells at 24 h post PEDV infection through transmission electron microscopy (TEM). A large number of double- or single-membrane vesicles were observed in PEDV-infected cells, which contained cytosolic components or sequestered organelles. However, autophagosome-like vesicles were rarely observed in the mock-infected cells (Figure 1A). It is well known that coronavirus infection can induce a large number of double-membrane vesicles (DMVs), and the functional link between autophagic DMVs and coronavirus-induced replication-associated DMVs remains controversial [22,23,24]. In this study, these two different DMVs might co-exist in PEDV-infected cells [25].

In addition, the GFP-LC3 tandem plasmid was transfected into Vero cells to verify the response of cellular autophagy to PEDV infection. As is well-known, the LC3, a protein, is selectively recruited to autophagic vesicles, which can be considered as its redistribution from a diffuse cytoplasmic localization to a distinctive punctate cytoplasmic pattern during autophagy [16]. GFP-LC3 positive cells treated with rapamycin showed high punctate LC3 accumulation. Additionally, large amounts of punctate GFP-LC3 proteins were observed in PEDV-infected cells at 18 hpi, while GFP-LC3 was detected as a diffuse distribution in mock-infected cells (Figure 1B), indicating that the accumulation of GFP-LC3 dots was induced by PEDV infection. The quantitative analysis of the percentage of punctate GFP-LC3 cells in the total GFP-positive cells was performed and 100 GFP-positive cells were detected in each sample. The percentage of punctate GFP-LC3 cells in PEDV infected cells was nearly 70%, which was obviously higher than that in the mock-treated cells (Figure 1C).

**Figure 2:**
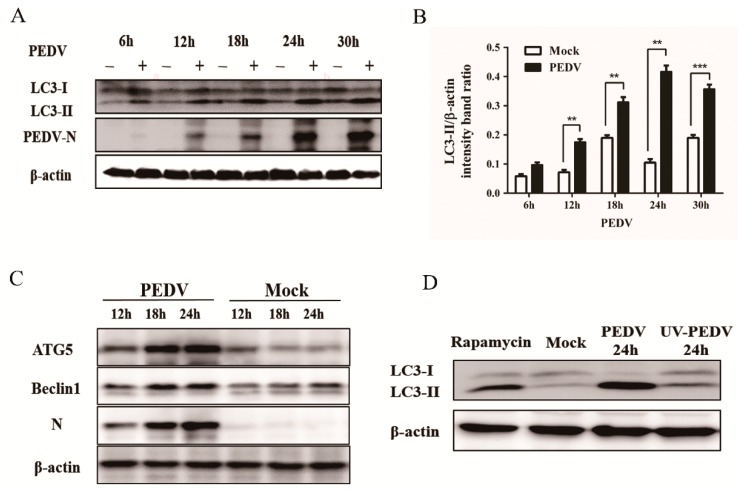
Expression of autophagy marker proteins in PEDV infected Vero cells. (**A**) Western blot analysis of the turnover of LC3-I to LC3-II in Vero cells at the indicated time points post PEDV infection using a polyclonal antibody against LC3 or a monoclonal antibody against PEDV N. β-actin expression was used as a protein loading control. (**B**) The intensity band ratio of LC3-II to β-actin was analyzed by using ImageJ software. The data were presented as mean ± SEM of three independent experiments (*t-*test, * *p* < 0.05, ** *p* < 0.01, *** *p* < 0.001). (**C**) Western blot analysis of the level of ATG5 and Beclin1 in Vero cells at 12, 18, and 24 hpi. β-actin expression was used as a protein loading control. (**D**) The turnovers of LC3-I to LC3-II were detected for mock-treated, rapamycin-treated, native PEDV, and UV-inactivated PEDV (MOI = 0.1) infection.

To further analyze whether autophagy was induced by PEDV infection, we examined the level of autophagy marker proteins in PEDV-infected cells by using immunoblotting. The conversion from LC3-I to LC3-II was monitored at 6, 12, 18, 24, and 30 h post PEDV infection. As shown in Figure 2A,B, a significant conversion of LC3-I to LC3-II was observed during the progression of PEDV infection, which was tracked by the PEDV N protein. Meanwhile, PEDV infection increased the expression of ATG5 and beclin1 in Vero cells relative to the mock-infected cells (Figure 2C). The results further supported that autophagy was induced by the PEDV infection.

Whether viral replication was required in PEDV-induced autophagy was confirmed by an experiment with ultraviolet (UV)-inactivated PEDV. The results demonstrated that no obvious detectable conversion from LC3-I to LC3-II was observed at 24 hpi, while autophagy was triggered normally by native PEDV (Figure 2D). The results indicated that viral replication was required for PEDV-induced autophagy.

### 3.2. PEDV Infection Can Enhance Autophagy Flux

**Figure 3:**
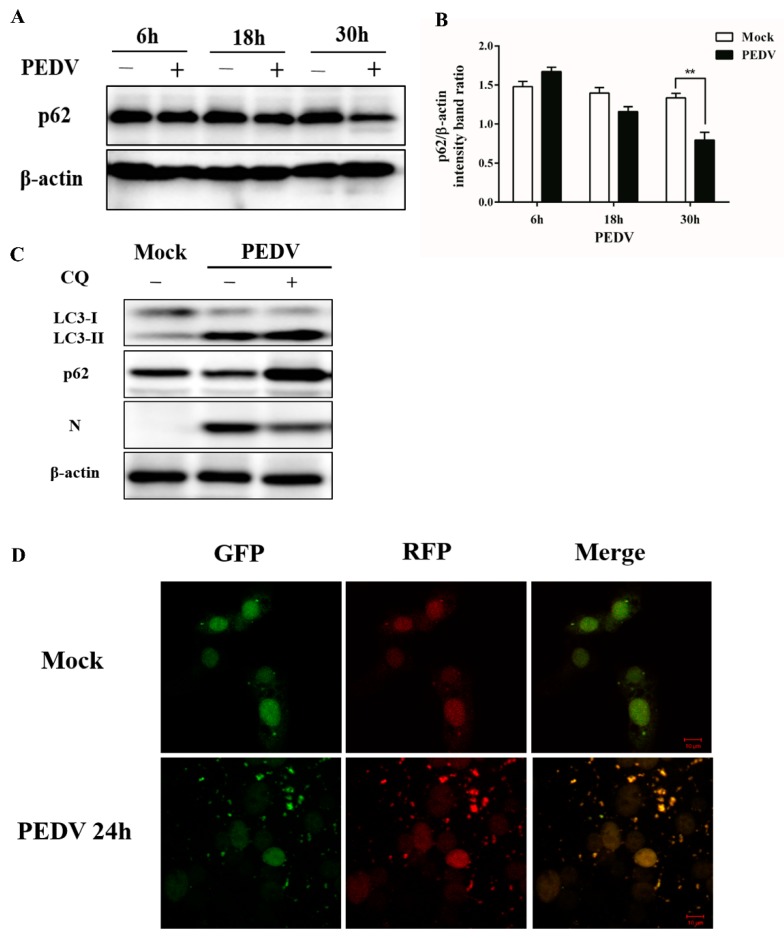
PEDV infection enhances autophagy flux. (**A**) Vero cells were mock-infected or infected with PEDV (0.1 MOI) for 6, 18, and 30 h. The cells were then analyzed by Western blot with antibodies against p62 and β-actin, separately. (**B**) The intensity band ratio of p62 to β-actin was analyzed by using ImageJ software. The data were presented as mean ± SEM of three independent experiments (*t*-test, * *p* < 0.05, ** *p* < 0.01, *** *p* < 0.001). (**C**) Vero cells were pretreated with CQ (50 μm) for 4 h, prior to PEDV (0.1 MOI) infection. After PEDV adsorption for 1 h, the cells were further cultured in fresh medium in the absence or presence of CQ. At 24 hpi, cell samples were detected by Western blot with antibodies against LC3, p62, N, and β-actin. (**D**) Vero cells were transfected with mRFP-GFP-LC3. Twenty-four hours later, the cells were mock-infected or infected with PEDV (0.1 MOI), then collected and visualized at 24 hpi, respectively. Scale bar, 10 μm.

The degradation of SQSTM1 (p62) was recognized as an indicator for assessing autophagy flux. Whether a complete autophagic process was triggered by the PEDV infection was first determined by the degradation of p62 through immunoblotting analysis. As shown in Figure 3A,B, p62 was slightly accumulated during the early life cycle of PEDV infection, but degraded at the later stages. Meanwhile, p62 showed no obvious change from 6 to 30 h in the mock-infected cells.

The autophagy flux upon PEDV infection was further verified by measuring the levels of LC3-II and p62 through the treatment with chloroquine (CQ), which can inhibit the fusion of the autophagosome with lysosome. As shown in Figure 3C, CQ elevated the levels of LC3-II and p62 markedly at 24 h post PEDV infection, compared to the mock-treated cells.

Furthermore, the PEDV-induced autophagy flux was also confirmed using a tandem-reporter construct, GFP-mRFP-LC3. GFP is sensitive to lysosomal proteolysis and may diminish quickly in acidic pH, whereas RFP (red fluorescent protein) retains its fluorescence even at acidic pH. Our results showed that PEDV infection resulted in a partially red fluorescence at 24 hpi (Figure 3D), indicating the elevated level of autophagic flux. Taken together, this substantial evidence suggests that PEDV infection can enhance autophagy flux in Vero cells.

### 3.3. Pharmacological Inhibition of Autophagy Decreases Viral Yield

**Figure 4:**
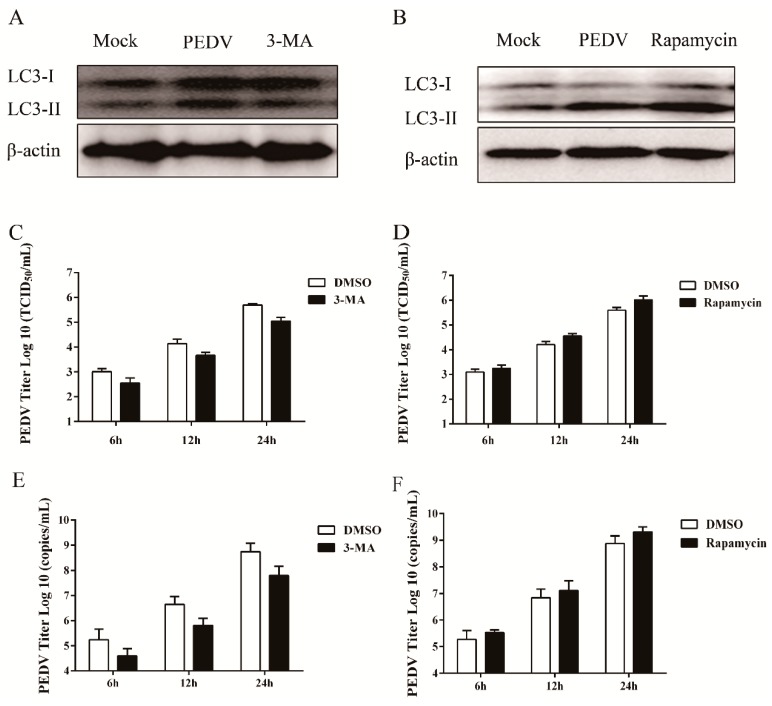
Pharmacological inhibition of autophagy decreases viral yield. (**A**,**B**) Vero cells were pretreated separately with 3-MA (5 mM) (**A**) or rapamycin (1 μg/mL) (**C**) for 4 h prior to PEDV (0.1 MOI) infection. After PEDV adsorption for 1 h, the cells were further cultured in fresh medium in the absence or presence of 3-MA or rapamycin. DMSO was used as a control. At 24 hpi, cell samples were detected by Western blot with antibodies against LC3 and β-actin. (**C**,**D**) The cells were collected separately at 6, 12, and 24 hpi to determine the viral titer. The data were presented as mean ± SEM of three independent experiments. (**E**,**F**) The cells were collected separately at 6, 12, and 24 hpi. The virus copy number was determined by real time PCR. The data were presented as mean ± SEM of three independent experiments.

The specific role of autophagy machinery on PEDV replication was explored by exposing Vero cells to 3-MA, which can inhibit autophagy at the early stage by suppressing the formation of autophagosomes [26]. As shown in Figure 4A,C,E, when compared to the mock-treated cells, 3-MA treatment not only reduced the level of LC3-II, but also significantly decreased the virus titer at different time points. In addition, CQ treatment (described above) reduced the expression of N protein, although it elevated the level of LC3-II (Figure 3C). These data indicated that autophagy inhibition could block PEDV infection. Similar results were also found in PEDV-infected ST cells.

Meanwhile, the role of autophagy machinery on PEDV replication was confirmed by rapamycin treatment, an inducer of autophagy through inhibition of the mTOR signaling pathway [8,17]. As shown in Figure 4B,D,F, induction of autophagy with rapamycin increased the LC3-II level and elevated the virus titer, compared to the mock-treated cells. The results suggested that autophagy induction could facilitate PEDV replication.

### 3.4. Silencing Endogenous Beclin1 or ATG5 Gene Reduces the PEDV Titer

**Figure 5:**
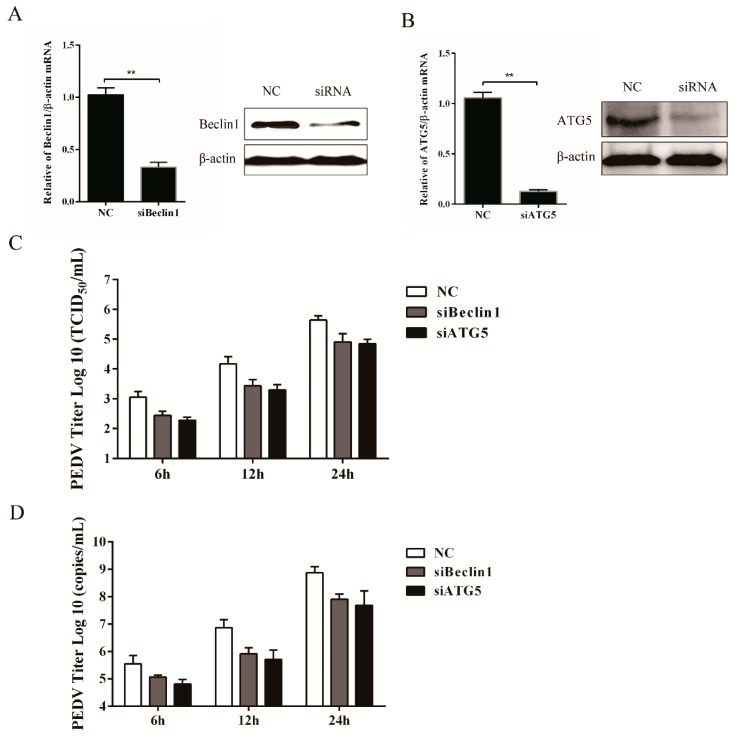
Inhibition of autophagy with specific siRNA targeting *Beclin1* or *ATG5* reduces PEDV replication. (**A**,**B**) Vero cells were transfected with siRNA targeting *Beclin1*, *ATG5*, or negative control (NC) for 48 h. The silencing efficiency was determined separately by quantitative real-time PCR and Western blot. (**C**) At 24 h post-transfection, cells were mock-infected or infected with PEDV for another 6, 12, and 24 h. The virus titer was determined by TCID_50_. The data were presented as mean ± SEM of three independent experiments. (**D**) The cells were treated as described in (**C**) and collected separately at 6, 12, and 24 hpi, respectively. The virus copy number was determined by qRT-PCR. The data were presented as mean ± SEM of three independent experiments.

The relationship between autophagy and PEDV replication was further confirmed through gene-silencing experiments, with the endogenous *Beclin1* or *ATG5* gene specifically silenced, which was verified at both the transcriptional and translational levels (Figure 5A,B). Data from Figure 5C,D demonstrated that suppression of Beclin1 or ATG5 expression obviously decreased the viral titer and virus copy number compared to the control group. All the aforementioned data indicated that the autophagy mechanism was triggered by PEDV infection to facilitate its replication.

### 3.5. Autophagy Has a Positive Correlation with NF-κB Signaling Pathway

**Figure 6:**
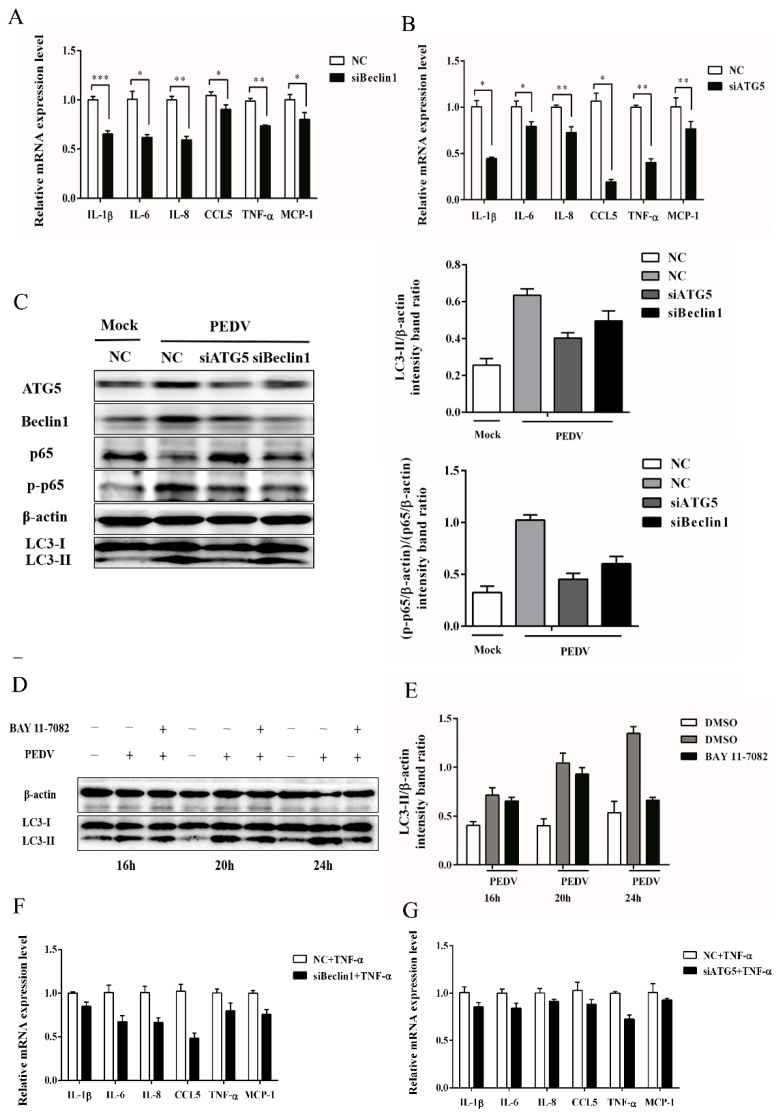
Autophagy mediates the production of inflammatory cytokines and correlates with the NF-κB signaling pathway in PEDV infected Vero cells. (**A**,**B**) The expression of inflammatory cytokines. Vero cells were transfected with siRNA targeting *Beclin1*, *ATG5*, or negative control (NC) for 24 h, followed by PEDV infection (0.1 MOI). The mRNA levels of cytokines were determined at 24 hpi by quantitative real-time PCR. (**C**) The level of LC3-II, p65, or phospho-p65 was also examined separately at 24 hpi with the corresponding antibodies by Western blot. The intensity band ratios of LC3-II to β-actin and p-p65 to p65 were analyzed by using ImageJ software. (**D**) Vero cells were pretreated with 10 μm BAY11-7082 for 12 h, then followed by PEDV infection for 16, 20, and 24 h, separately. The cell samples were collected for LC3-II detection by Western blot. (E) The intensity band ratio of LC3-II to β-actin was analyzed by using ImageJ software. All data were presented as mean ± SEM of three independent experiments. (**F**,**G**) Vero cells were transfected with siATG5, siBeclin1, or negative control. Twenty-four hours post-transfection, the cells were treated with TNF-α for 4 h, and then the expression of inflammatory cytokines were determined by qRT-PCR. The data were presented as mean ± SEM of three independent experiments.

Accumulating data revealed a strong association between autophagy and the host immune response in the progression of virus infection [27,28]. Thus, the role of autophagy in PEDV induced inflammatory responses was investigated first by measuring the level of inflammatory cytokines under the circumstances when autophagy was suppressed by silencing the expression of Beclin1 and ATG5 proteins. Data showed that the inflammatory cytokines (such as IL-1β, IL-6, IL-8, CCL5, TNF-α, and MCP-1) were significantly down-regulated at 24 hpi when autophagy was inhibited in PEDV infected cells (Figure 6A,B), suggesting the potential involvement of autophagy in PEDV induced inflammation. It is well-known that the NF-κB signaling pathway plays a pivotal role in PEDV induced inflammatory response and Vero cells are interferon deficient [19,29]. Therefore, the influence of autophagy on the NF-κB pathway was determined under the deficient expression of Beclin1 and ATG5 at 24 h upon PEDV infection by immunoblotting analysis. The level of LC3-II decreased, and the phosphorylation of p65 was also down-regulated at the same time (Figure 6C). The results indicated that autophagy might participate in PEDV induced inflammation through the NF-κB pathway.

To further explore the relationship between autophagy and the NF-κB pathway mediated inflammatory response, the NF-κB pathway was attenuated by administrating BAY 11-7082, an NF-κB inhibitor. As shown in Figure 6D,E, the administration of BAY 11-7082 abolished the elevated level of LC3-II at 16, 20, and 24 h post PEDV infection, which was especially significantly at 24 hpi. These data indicated that a potential positive feedback loop between autophagy and the NF-κB signaling pathway might exist during PEDV infection.

Considering that autophagy inhibition can block PEDV replication, a TNF-α induction of inflammatory cytokines experiment in autophagy-deficiency cells was carried out to exclude the effect of PEDV replication on cytokine production. From Figure 6F,G, it can be seen that the expression of inflammatory cytokines induced by TNF-α in ATG5-or Beclin1-deficient cells was significantly down-regulated, when compared to the transfected cells of the negative control. These results also supported the potential positive relationship between autophagy and the NF-κB signaling pathway induced by PEDV infection.

### 3.6. Pharmacological Regulation of Autophagy Does Not Affect Cell Viability

**Figure 7:**
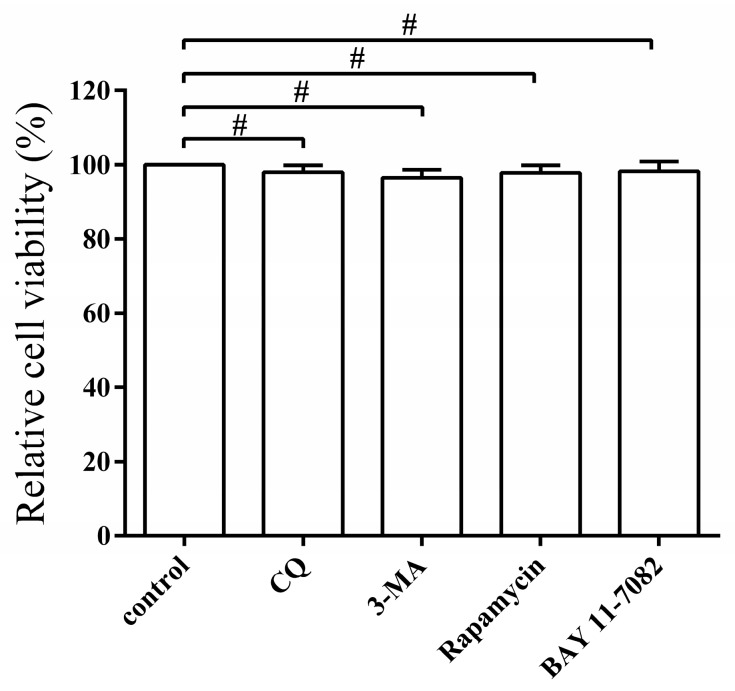
Pharmacological regulation of autophagy does not affect cell viability. The cell viability was determined by MTT assay after treatment with CQ (50 μm), 3-MA (5 μm), rapamycin (1 μg/mL), and BAY 11-7082 (10 μm) for 48 h, respectively. The data represent the mean ± SEM of three independent experiments. (*t*-test, ^#^ *p* > 0.05).

The effect of the autophagy regulators on the capability of PEDV replication by changing the cell viability was tested by the MTT assay. No significant effects on cell viability were observed from the treatment with CQ, 3-MA, rapamycin, or BAY11-7082, respectively (*p* > 0.05) (Figure 7), indicating that pharmacological regulation of autophagy does not affect the cell viability.

## 4. Discussion

In recent years, autophagy has been widely investigated due to its important role in the pathogenesis of many diseases [9,30]. The relationship between autophagy and viral infection, including coronavirus, has attracted the attention of an increasing number of researchers [11,31,32]. For instance, the formation of double membrane-bound MHV replication complexes was found essential for the autophagy induced by mouse hepatitis virus (MHV) infection [33]. A further study revealed that the autophagy-like process induced by infectious bronchitis virus (IBV) infection might not be important for virus replication [34]. In addition, autophagy was confirmed to negatively regulate transmissible gastroenteritis virus (TGEV) replication [35]. However, the specific role of autophagy on PEDV infection has not been reported until now. In this study, we provide the first strong evidence that PEDV infection can trigger autophagy to facilitate its replication.

In the present study, we firstly found that PEDV infection promoted the formation of DMVs by TEM, which is a hallmark of coronavirus replication and might potentially provide a platform for viral RNA synthesis [23,36]. In coronavirus infection, the formation of DMVs is usually believed to be associated with the autophagy pathway. This observation was further supported by the accumulation of GFP-LC3 dots in virus induced syncytia and the increased expression of autophagic marker proteins from the immunoblotting analysis. These data indicated that autophagy activity might be triggered by PEDV infection [37]. In addition, autophagy was documented to play a role in syncytia formation, which could further increase autophagy in multinucleated cells as well. Therefore, we inferred that autophagy might closely associate with the syncytia formation in Vero cells during PEDV infection [38,39]. Moreover, the UV inactivation test implied that the effective replication of PEDV was required for autophagy induction. Previous reports indicated that autophagosome accumulation might be attributed to their de novo formation or a block in trafficking to lysosomes for maturation. The cellular state of autophagy can be measured by detecting the degradation of SQSTM1 (p62), using a lysosome inhibitor, or a tandem reporter construct mRFP-GFP-LC3 [40]. Our results from these tests indicated that a complete autophagy process might be triggered to modulate PEDV infection in Vero cells.

Recent studies have demonstrated that the replication mechanisms may vary among different members of the *Coronaviridae* family. For example, TGEV infection induces autophagy to negatively regulate its replication [35]. IBV can induce autophagy but does not require autophagy for its replication [34]. The specific effect of autophagy induction on MHV infection is controversial. Prentice et al. reported that MHV utilized the autophagy process to form DMVs to enhance its replication [33]. However, another study showed that autophagy was not important for MHV replication [41]. In the present study, the concrete effect of autophagy upon PEDV infection was examined by administrating pharmacological regulators. The yield of PEDV was found to be suppressed whenever the autophagy process was inhibited by 3-MA at the early stage, or by CQ at the late stage. Additionally, the induction of autophagy by rapamycin also enhanced the viral titer. The effect of autophagy modulation on PEDV replication was further evaluated by silencing the two essential endogenous components *Beclin1* and *ATG5* [26,42]. Beclin1 (ATG6), a critical component in the class III PI3 kinase complex (PI3KC3), is involved in the initial step of autophagosome formation, while the ATG12-ATG5 conjugate, a key regulator, participates in autophagosome maturation [43]. The abolishment of their expression reduced the PEDV titer. These results suggested that autophagy induction might benefit PEDV replication. Autophagy has also been documented to have a complex interaction with apoptosis during viral infection [44,45,46]. For instance, influenza A virus was documented to induce apoptosis in autophagy protein deficient cells [47]. Autophagy was also reported to postpone apoptotic cell death during porcine reproductive and respiratory syndrome virus (PRRSV) infection through Bad-Beclin1 interaction [48,49]. The interplay of autophagy and apoptosis during PEDV infection needs to be further elucidated for a better understanding of the autophagy mechanism by which PEDV benefits its infection.

Recently, autophagy has been identified as an important component of the immune system, functioning from the elimination of infectious agents to the modulation of inflammatory responses [50,51]. The crosstalk between autophagy and inflammation has drawn much attention from researchers in recent years, while the relationship between autophagy and PEDV induced inflammatory responses is largely unclear. In the present study, we showed the expression of inflammatory cytokines and the activation of the NF-κB signaling pathway were both restrained in PEDV infected autophagy deficient cells, implying that autophagy might participate in PEDV induced inflammatory responses, especially for the NF-κB signaling pathway. Meanwhile, Vero cells with a NF-κB pathway deficiency exhibited a decreased level of LC3-II after PEDV infection, suggesting that the NF-κB signaling pathway might also have a positive role in autophagy induction. It was worth mentioning that the PEDV yield was reduced in both autophagy deficient or NF-κB pathway deficient cells. The decreased expression of inflammatory cytokines induced by TNF-α in autophagy deficient cells might provide new clues for deciphering the complex relationship between autophagy and inflammation. The cause-and-effect relationship between the restriction of PEDV yield reduction on the expression of inflammatory cytokines and the restraint of inflammation inhibition on PEDV replication in autophagy deficient cells needs further elucidation. A previous study reported that the positive feedback loop between autophagy and the NF-κB signaling cascade might exacerbate the inflammation induced by H5N1 pseudotyped viral particles [28]. The inhibition of NF-κB was also documented to induce autophagy suppression, leading to apoptosis in pancreatic ductal adenocarcinoma cells [52]. We speculate that autophagy and inflammatory cascade reactions might be positively regulated by PEDV infection to exacerbate inflammation. Furthermore, apoptosis might play a pivotal role in the complex relationship between autophagy and inflammation.

In summary, this is the first report concerning the potential induction of autophagy in PEDV infected Vero cells to facilitate its replication. Autophagy induction might be manipulated by PEDV to mediate the inflammatory cascade responses, and have a positive feedback loop with the NF-κB signaling pathway. Our knowledge regarding the interplay between PEDV infection and autophagy is still insufficient. The integrated data facilitate our understanding of the pathogenesis of PEDV infection and provide novel insights into the development of effective therapeutic strategies. Further research can focus on the complex relationship between autophagy modulation and immune responses, as well as apoptosis.

## Figures and Tables

**Table 1:** Primers used for real-time real time PCR.

Primer	Sequence (5’–3’)
PEDV-F	CGTACAGGTAAGTCAATTAC
PEDV-R	GATGAAGCATTGACTGAA
PEDV-probe-M	TTCGTCACAGTCGCCAAGG
ATG5-F	TTCACGCTATATCAGGAT
ATG5-R	ATCTCACTAATGTCTTCTTG
Beclin1-F	TGGCACAATCAATAACTTC
Beclin1-R	CAAGCAGCATTAATCTCAT
IL-6-F	TGTGAAAGCAGCAAAGAG
IL-6-R	AGTGTCCTCATTGAATCCA
IL-1β-F	GCGGCAACGAGGATGACTT
IL-1β-R	TGGCTACAACAACTGACACGG
IL-8-F	GGAACCATCTCGCTCTGTGTAA
IL-8-R	GGTCCACTCTCAATCACTCTCAG
CCL5-F	ACGCCTCGCTGTCATCCT
CCL5-R	GCACTTGCCACTGGTGTAGAA
TNF-α-F	CACCACGCTCTTCTGTCT
TNF-α-R	AGATGATCTGACTGCCTGAG
MCP-1-F	CTTCTGTGCCTGCTGCTCATA
MCP-1-R	ACTTGCTGCTGGTGATTCTTCT
GAPDH-F	ACATCATCCCTGCCTCTACTG
GAPDH-R	CCTGCTTCACCACCTTCTTG
β-actin-F	TTAGTTGCGTTACACCCTTTC
β-actin-R	ACCTTCACCGTTCCAGTT
